# Reply to Berk et al. Comment on “Moriconi et al. Very-Low-Calorie Ketogenic Diet as a Safe and Valuable Tool for Long-Term Glycemic Management in Patients with Obesity and Type 2 Diabetes. *Nutrients* 2021, *13*, 758”

**DOI:** 10.3390/nu13103639

**Published:** 2021-10-18

**Authors:** Eleonora Moriconi, Elisabetta Camajani, Andrea Fabbri, Andrea Lenzi, Massimiliano Caprio

**Affiliations:** 1 Laboratory of Cardiovascular Endocrinology, IRCCS San Raffaele Pisana, 00166 Rome, Italy; eleonoramoriconi87@gmail.com; 2 PhD Programme in Endocrinological Sciences, Sapienza University of Rome, 00161 Rome, Italy; elisabetta.camajani@uniroma5.it; 3 Department of Human Sciences and Promotion of the Quality of Life, San Raffaele Roma Open University, 00166 Rome, Italy; 4 Division of Endocrinology, CTO Andrea Alesini Hospital, ASL Roma 2, Department of Systems Medicine, University of Rome “Tor Vergata”, 00133 Rome, Italy; andrea.fabbri@uniroma2.it; 5 Section of Medical Pathophysiology and Endocrinology, Department of Experimental Medicine, Sapienza University of Rome, 00161 Rome, Italy; andrea.lenzi@uniroma1.it

We thank the authors of the comment [1] for their expressed interest in our article [2].

It is true that ketogenic diets significantly differ in macronutrient composition [3], encompassing different dietary models that can achieve various degrees of nutritional ketosis. This is why such nutritional approach represents a viable tool in different clinical contexts, ranging from neurologic diseases to metabolic derangements. However, in our work, we clearly defined the characteristics of the ketogenic diet used in the study. Nowadays, very low calorie ketogenic diets (VLCKD) represent a validated approach in the context of obesity and type 2 diabetes, and authoritative scientific societies (Italian Society of Endocrinology, European Association for the Study of Obesity) have published recent position statements clearly defining their characteristics in terms of nutrient composition and temporal structure [4,5]. Moreover, a consensus report on nutrition therapy from the American Diabetes Association suggested very low carbohydrate diets for patients with type 2 diabetes not meeting glycemic targets, or requiring a reduction in antiglycemic medications [6].

In the Materials and Methods section of the paper, we did provide a clear description of the nutritional intervention, characterizing macronutrient and micronutrient composition in the different phases during the study period, and highlighting that the length of VLCKD phases was personalized according to the weight loss target. Capillary blood ketone levels were monitored during the first 3 months from the beginning of the nutritional intervention (i.e., during the ketogenic phases and at the first visit of the re-educational stage) and ranged between 0.5 and 1.2 mmol/L in the active ketosis phases, reaching lower levels after the reintroduction of carbohydrates. Therefore, we did confirm a mild ketosis state during the nutritional intervention with VLCKD, in line with previous reports using the same nutritional approach [7]. Patients who did not reach and maintain the minimum threshold value for nutritional ketosis (0.5 mmol/L) were not selected for the study.

Data from patients eligible for the study were collected during a time period spanning from April 2018 to June 2020. We agree with the authors about the relevant differences in baseline anthropometric measures between the study groups; this aspect has been, in fact, reported as a limitation of the study. Given that subjects were free to choose the dietary intervention, it is not surprising that patients affected by more severe degrees of obesity were more likely to accept and adhere to a nutritional regimen with a higher promise for rapid weight loss.

However, we believe that this aspect does not jeopardize the significance of our work, which was based on a retrospective observational study, and therefore should be considered as a simple observation of a real-world clinical practice setting, evaluating the efficacy of different nutritional strategies for the management of patients with obesity and type 2 diabetes. On the other hand, diabetic patients are known to adhere poorly to nutritional interventions and lifestyle modifications, irrespective of their body mass index, with obese subjects showing even less adherence and motivation to improve their health status.

However, we fully agree with the authors on the consideration that adequately controlled clinical trials are necessary to draw solid conclusions on this delicate issue and to confirm our data.

## Data Availability

Data sharing is not applicable to this article.
